# Perioperative TAS-118 plus oxaliplatin in patients with locally advanced gastric cancer: APOLLO-11 study

**DOI:** 10.1007/s10120-023-01388-z

**Published:** 2023-04-08

**Authors:** Daisuke Takahari, Hitoshi Katai, Atsuo Takashima, Naoki Izawa, Naoki Ishizuka, Manabu Ohashi, Shinya Mikami, Takeru Wakatsuki, Izuma Nakayama, Keisho Chin, Satoshi Ida, Koshi Kumagai, Souya Nunobe, Satoru Iwasa, Hirokazu Shoji, Takeyuki Wada, Ayako Doi, Takaki Yoshikawa, Takeshi Sano, Narikazu Boku, Kensei Yamaguchi

**Affiliations:** 1 Department of Gastroenterological Chemotherapy, Cancer Institute Hospital of Japanese Foundation for Cancer Research, 3-8-31 Ariake, Koto-ku, Tokyo, 135-8550 Japan; 2 Department of Gastric Surgery, National Cancer Center Hospital, Tokyo, Japan; 3 Department of Gastrointestinal Medical Oncology, National Cancer Center Hospital, Tokyo, Japan; 4 Department of Clinical Oncology, St. Marianna University School of Medicine, Kawasaki, Japan; 5 Department of Clinical Planning and Strategy, Cancer Institute Hospital of the Japanese Foundation for Cancer Research, Tokyo, Japan; 6 Department of Gastroenterological Surgery, Cancer Institute Hospital of the Japanese Foundation for Cancer Research, Tokyo, Japan; 7 Department of Gastrointestinal and General Surgery, St. Marianna University School of Medicine, Kawasaki, Japan

**Keywords:** Gastric cancer, Gastroesophageal junction cancer, Perioperative chemotherapy, TAS-118, Oxaliplatin

## Abstract

**Background:**

We investigated the feasibility of perioperative chemotherapy with S-1 and leucovorin (TAS-118) plus oxaliplatin in patients with locally advanced gastric cancer.

**Methods:**

Patients with clinical T3–4N1–3M0 gastric cancer received four courses of TAS-118 (40–60 mg/body, orally, twice daily for seven days) plus oxaliplatin (85 mg/m^2^, intravenously, day one) every two weeks preoperatively followed by gastrectomy with D2 lymphadenectomy, followed by postoperative chemotherapy with either 12 courses of TAS-118 monotherapy (Step 1) or eight courses of TAS-118 plus oxaliplatin (Step 2). The primary endpoints were completion rates of preoperative chemotherapy with TAS-118 plus oxaliplatin and postoperative chemotherapy with TAS-118 monotherapy (Step 1) or TAS-118 plus oxaliplatin (Step 2).

**Results:**

Among 45 patients enrolled, the preoperative chemotherapy completion rate was 88.9% (90% CI 78.0–95.5). Major grade ≥ 3 adverse events (AEs) were diarrhoea (17.8%) and neutropenia (8.9%). The R0 resection rate was 95.6% (90% CI 86.7–99.2). Complete pathological response was achieved in 6 patients (13.3%). Dose-limiting toxicity was not observed in 31 patients receiving postoperative chemotherapy (Step 1, n = 11; Step 2, n = 20), and completion rates were 90.9% (95% CI 63.6–99.5) for Step 1 and 80.0% (95% CI 59.9–92.9) for Step 2. No more than 10% of grade ≥ 3 AEs were observed in  patients receiving Step 1. Hypokalaemia and neutropenia occurred in 3 and 2 patients, respectively, receiving Step 2. The 3-year recurrence-free and overall survival rates were 66.7% (95% CI 50.9–78.4) and 84.4% (95% CI 70.1–92.3), respectively.

**Conclusions:**

Perioperative chemotherapy with TAS-118 plus oxaliplatin with D2 gastrectomy is feasible.

**Supplementary Information:**

The online version contains supplementary material available at 10.1007/s10120-023-01388-z.

## Introduction

Gastric cancer is the fifth most common malignancy and the third leading cause of cancer death worldwide [1]. However, perioperative chemotherapy has improved the long-term outcomes of patients with locally advanced resectable gastric cancer (LAGC) [2].

The Japanese gastric cancer treatment guidelines recommend D2 gastrectomy and postoperative chemotherapy for LAGC [3], although the treatment outcomes have remained unsatisfactory. One option for improving the outcomes of patients with LAGC has been the intensification of postoperative chemotherapy; however, the compliance has generally been low [4]. In contrast, preoperative chemotherapy has allowed better compliance than postoperative chemotherapy. Perioperative 5-FU, leucovorin, oxaliplatin, and docetaxel (FLOT) have shown significant improvement in overall survival (OS) compared with epirubicin, oxaliplatin, and capecitabine [5]. A Korean phase 3 study adding preoperative chemotherapy with docetaxel, oxaliplatin, and S-1 (DOS) to postoperative chemotherapy with S-1 improved relapse-free survival (RFS) [6]. Furthermore, a phase 3 study in China showed that perioperative S-1 plus oxaliplatin (SOX) improved RFS [7]. Therefore, preoperative chemotherapy may be a more effective treatment for LAGC.

TAS-118 (Taiho Pharmaceutical, Tokyo, Japan) is a novel oral antitumour agent that combines S-1 (tegafur, gimeracil, and oteracil potassium) with leucovorin (LV) in granules [8]. LV is a biochemical modulator that enhances the antitumour effect of 5-FU by establishing a ternary complex with thymidylate synthase, fluorodeoxyuridine monophosphate, and 5,10-methylenetetrahydrofolate, thereby strongly inhibiting DNA synthesis [9]. A meta-analysis of patients with colorectal cancer showed that 5-FU and LV increased the response rate (RR) to treatment and improved OS compared with 5-FU alone [10]. A randomized phase 3 study of patients with AGC in Japan and Korea found that TAS-118 plus oxaliplatin provided a higher response to treatment than S-1 plus cisplatin (SP) (73% vs. 50%, respectively), and prolonged progression-free survival (PFS) (median 7.1 months vs. 6.4 months, respectively; HR 0.79, 95% CI 0.66–0.93) and OS (median 16.0 months vs. 15.1 months respectively; HR 0.83, 95% CI 0.69–0.99) [11]. Altogether, the results suggest that TAS-118 may be promising in the perioperative setting for patients with LAGC.

This study aimed to investigate the feasibility of perioperative TAS-118 plus oxaliplatin in patients with LAGC and clinically diagnosed lymph node metastases.

## Patients and methods

### Study design

**Fig. 1:**
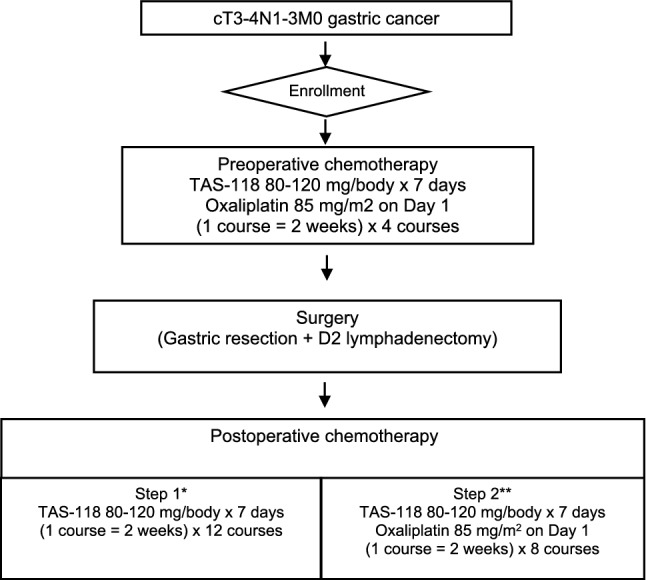
Study design. (One asterisk) Check the number of DLTs in the first 10 pts of 2 courses of postoperative TAS-118. If 5 subjects are confirmed to have not developed DLT, the study will advance to Step 2. (Two asterisks) If 6 of 10 subjects are confirmed to have developed DLT, then the study will go back to Step 1. Subject enrollment in the selected step will continue until 20 subjects have been enrolled

This was an investigator-initiated, multicentre, open-label, non-randomized study that was designed to investigate the safety and efficacy of TAS-118 plus oxaliplatin as preoperative chemotherapy and the feasibility of TAS-118 monotherapy (Step 1) and TAS-118 combined with oxaliplatin (Step 2) as postoperative chemotherapy (Fig. 1).

The study was conducted at three sites in Japan. The protocol was approved by the institutional review board at each participating centre and conducted in accordance with good clinical practice. Written informed consent was obtained from all participants before enrolment. An independent data-monitoring committee (IDMC) monitored trial safety and performance. This trial is registered on UMIN000024688.

### Patients

The enrolment criteria were as follows: patient age 20–79 years; Eastern Cooperative Oncology Group performance status (PS) 0–1; histologically confirmed adenocarcinoma of the stomach and/or gastroesophageal junction (GEJ); clinically diagnosed T3–4, N1–3, and M0 disease according to the TNM-7th/Japanese Classification of Gastric Carcinoma (JGCA) 14th Edition; and adequate organ function. Lymph nodes ≥ 8 mm in the short axis or ≥ 10 mm in the long axis were considered metastatic. The study protocol contains comprehensive descriptions of the inclusion and exclusion criteria.

### Study procedures

The 3-part treatment protocol was as follows: (1) preoperative chemotherapy with four courses of TAS-118 and oxaliplatin, (2) gastrectomy with D2 lymphadenectomy, (3) postoperative chemotherapy with 12 courses of TAS-118 monotherapy (Step 1) or eight courses of TAS-118 plus oxaliplatin (Step 2).

For preoperative chemotherapy, TAS-118 (40–60 mg/body according to body surface area) was administered orally twice a day on days 1–7, followed by a 1-week rest. Oxaliplatin (85 mg/m^2^) was administered intravenously on day 1 every two weeks.

Gastrectomy with D2 lymph node dissection was performed within 56 days after the last dose of preoperative chemotherapy. Resection criteria after preoperative chemotherapy were (1) R0 resection by gastrectomy with D2 lymph node dissection considered possible based on computed tomography (CT) findings and (2) white blood cell and platelet counts ≥ 3000/mm^3^ and ≥ 100,000/mm^3^, respectively.

Postoperative chemotherapy was started within 56 days after a pathologically-confirmed R0 resection. Postoperative chemotherapy was omitted for patients achieving pathological complete response (pCR) with no involved lymph nodes (ypT0N0). The doses and schedules of TAS-118 with/without oxaliplatin were the same as for preoperative chemotherapy. All criteria for starting pre- and postoperative chemotherapy and for reducing the doses of TAS-118 and oxaliplatin are in the study protocol.

Each patient underwent a physical examination and laboratory testing within seven days before initiating pre and postoperative chemotherapy and one day before or on the day of each subsequent course of chemotherapy. Adverse events (AEs) were evaluated according to the National Cancer Institute Common Terminology Criteria for AEs (CTCAE), version 4.03. Surgical complications were assessed by CTCAE, version 4.03 and the Clavien-Dindo classification. Tumour responses during preoperative chemotherapy were evaluated after two and four courses by each investigator according to the Response Evaluation Criteria in Solid Tumours (RECIST), version 1.1.

Pathological evaluations, including depth of primary tumour (T), lymph node involvement (N), and resection status (RX, R0, or R1), were performed by the institutional pathologist according to the TNM-7th/JGCA 14th Edition. The pathological response was evaluated according to the Japanese Classification of Gastric Carcinoma [12] and Becker’s criteria [13]. The pathological response rate (pRR) was defined as viable tumour cells remaining in no more than 2/3 of the pre-existing tumour area (Grade 1b, 2, 3), including pathological complete response (pCR), defined as no residual tumour cells (Grade 3).

### Two-step procedure to evaluate postoperative chemotherapy protocols

Although completion rates of postoperative chemotherapy are considered an essential measure of feasibility, assessment of treatment completion for each patient would be time consuming. Therefore, the decision for proceeding to Step 2 was based on the occurrence of dose-limiting toxicities (DLTs) during the first 2 courses of postoperative chemotherapy. The study was planned to proceed to Step 2 if 5 or fewer of the first 10 patients receiving postoperative chemotherapy in Step 1 developed DLTs but would not proceed if more than 5 patients developed DLTs.

For the Step 2 protocol, if 5 or fewer of the first 10 patients developed DLTs, Step 2 chemotherapy would be continued for up to a total of 20 patients, but if more than 5 patients developed DLTs, Step 2 would be discontinued. Step 1 would then be restarted for up to a total of 20 patients, which would include the first 10 patients in Step 1. Please refer to the study protocol for the details of the step-wise proceedings.

DLTs were defined as the occurrence of any of the following protocol-treatment–related AEs: (1) Grade ≥ 3 nausea/vomiting/anorexia/diarrhoea/mucositis/fatigue lasting longer than seven days despite adequate treatment, (2) Grade 3 non-haematological toxicity requiring discontinuation of treatment, (3) any toxicity leading to administration of TAS-118 for less than 5 days, (4) Grade 4 non-haematological toxicity, (5) second course of TAS-118 delayed for more than 15 days, (6) discontinuation of oxaliplatin due to any AE except anaphylaxis (Step 2 only).

### Endpoints and assessment

The primary endpoints were as follows: (1) completion rate of preoperative chemotherapy with TAS-118 plus oxaliplatin and gastrectomy for all patients, (2) completion rate of postoperative chemotherapy with TAS-118 monotherapy and TAS-118 plus oxaliplatin in patients undergoing postoperative chemotherapy. The secondary endpoints included the completion rate of both preoperative chemotherapy and surgery, relative dose intensity (RDI), clinical and pathological response rates to preoperative chemotherapy, R0 resection rate, downstaging rate, RFS, OS, and safety.

### Definition of the completion of treatment and RDI

The completion of treatment in each part of the protocol was defined as follows: (1) preoperative TAS-118 chemotherapy was administered through 4 courses, even if the administration of oxaliplatin was skipped or discontinued per protocol, (2) surgical procedure leading to R0 or R1(CY+) resection, (3) postoperative TAS-118 chemotherapy was administered through all 12 courses for Step 1 and all 8 courses for Step 2, even if oxaliplatin was skipped or discontinued per protocol. The RDI is the ratio of the cumulative dose in mg of each drug that was actually administered to the planned cumulative dose in mg of each drug that would have been administered assuming treatment was not suspended and the dose was not reduced.

### Statistical analysis

Because the feasibility of TAS-118 and TAS-118 plus oxaliplatin had already been confirmed in patients with AGC [11], the feasibility of these agents in postoperative chemotherapy was the major study focus. Our study was not designed to evaluate MTDs based on DLT incidence, as in conventional phase I studies. If 6 or more of the initial 10 patients in each step experienced DLT, the lower limit of the 95% CI would be higher than 30%; this decision rule means that we could not accept a 30% or higher incidence of DLT. Finally, if at least 9 or more of 20 patients in each step developed DLTs, the study power would be 76.2% with an alpha error of 13.1% for deciding that postoperative chemotherapy in the corresponding step would not be feasible, with the expectation of a DLT occurrence rate of 35% (threshold 55%).

With an expected completion rate of 65% (threshold 45%) for postoperative chemotherapy, with reference to previous trials (FLOT4 study [5]: 37–46%, CLASSIC study [14]: 67%), 20 patients completing each step would provide a study power of 60.1%, with an alpha error of 5.8%. Considering that 20 patients would be required for each step, and approximately 10% of patients might not receive postoperative chemotherapy for any reason, the sample size was set at 45 patients. Additionally, 45 patients would provide a study power of 96.8% with an alpha error of 4.71%, with the expectation of a completion rate of 90% (threshold 70%) with reference to a previous trial (FLOT4 study [5]: 90–91%) for preoperative chemotherapy.

An interim analysis was planned at the time of the evaluation of DLT for the first 10 patients undergoing Steps 1 and 2. Descriptive statistical analyses were performed to provide statistical results and confidence intervals (CIs). CIs were set at 90% for the completion rate, objective response rate, R0 resection rate, pathological response rate and downstaging rate and at 95% for the OS and RFS.

OS and RFS were estimated by the Kaplan–Meier method. OS was defined as the time from enrolment until death from any cause. RFS was defined as the time from the date of enrolment to the first date of relapse or death from any cause, whichever comes first, if a patient received curative surgery. For patients who did not undergo curative surgery, the time point at which it was determined that surgery would not be conducted was adopted as the event. Survival and safety were assessed in all patients and are in the full analysis set. SAS ver. 9.4 (SAS Institute, Cary, NC, USA) was used for the analysis.

## Results

### Patients

**Fig. 2:**
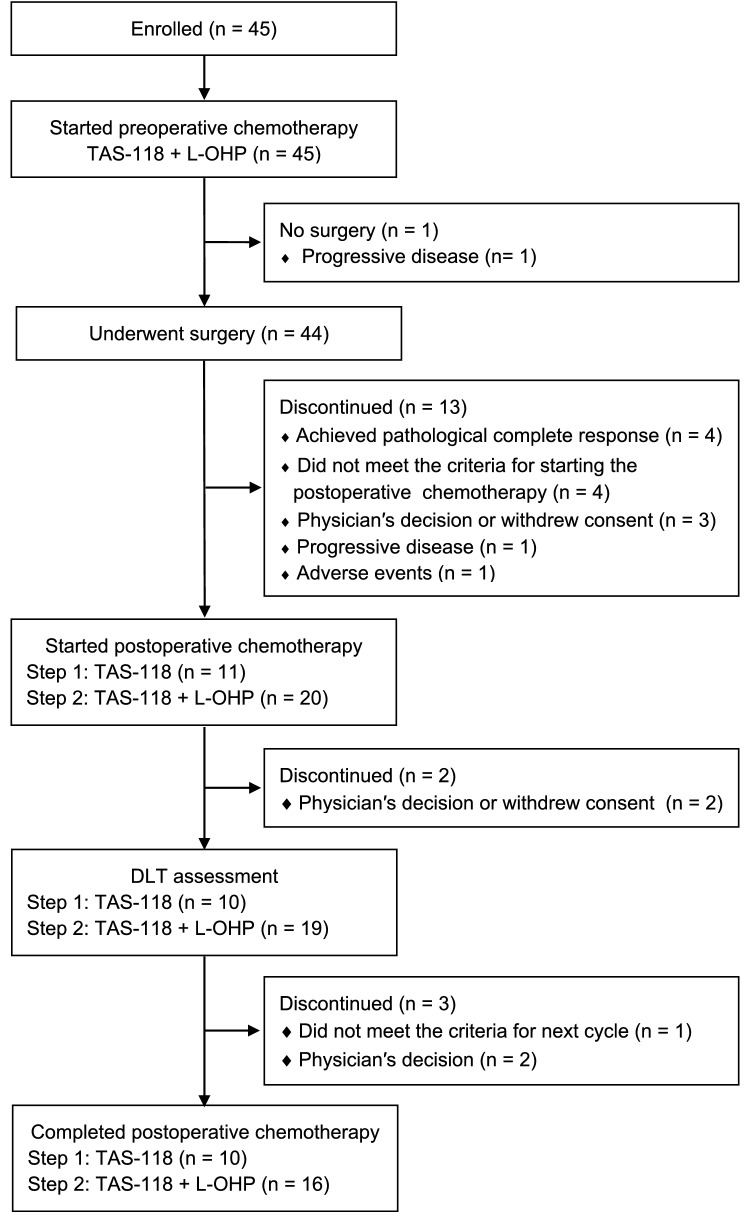
Trial profile. *TAS-118* S-1 plus leucovorin, *L-OHP* oxaliplatin, *DLT* dose limiting toxicity

Between December 2016 and February 2019, 45 patients were enrolled and received preoperative chemotherapy, and 44 then underwent surgery. A total of 31 patients (11 for Step 1 and 20 for Step 2) started postoperative chemotherapy (Fig. 2).

Table 1 shows the patients’ baseline characteristics. The stomach was the site of the primary tumour in 40 of 45 patients (88.9%). The histological type was predominantly intestinal (n = 31, 68.9%). The clinical stages in the 45 patients were IIB (n = 11, 24.4%), IIIA (n = 16, 35.6%), IIIB (n = 15, 33.3%), and IIIC (n = 3, 6.7%).

**Table 1:** Patient demographics and clinical characteristics

Characteristics	Total (N = 45)
*Age at enrollment, years*	
Median [range]	64 [32–78]
*Gender*	
Male	37 (82.2)
Female	8 (17.8)
*ECOG PS*	
0	38 (84.4)
1	7 (15.6)
*Primary site*	
Gastric	40 (88.9)
EGJ	5 (11.1)
*Histrogy*	
Intestinal	31 (68.9)
Diffuse	14 (31.1)
*Clinical T stage*	
3	13 (28.9)
4a	32 (71.1)
*Clinical N stage*	
1	25 (55.6)
2	17 (37.8)
3	3 (6.7)
*Clinical stage*	
IIB	11 (24.4)
IIIA	16 (35.6)
IIIB	15 (33.3)
IIIC	3 (6.7)

### Preoperative chemotherapy

Forty of 45 patients completed preoperative chemotherapy (completion rate of 88.9% [90% CI 78.0–95.5]). Three patients discontinued treatment because of AEs, including elevated AST (n = 1), decreased appetite and diarrhoea (n = 1), and duodenal perforation (n = 1). The duodenal perforation occurred at a distance from the tumour after 2 courses of treatment and might have been due to chemotherapy. One patient showed disease progression, and one withdrew consent for therapy. The major Grade 3 or 4 AEs consisted of diarrhoea (17.8%), neutrophil count decreased (8.9%), decreased appetite (4.4%), and stomatitis (4.4%) (Table 2). The median RDI was 86.0% for TAS-118 and 90.6% for oxaliplatin.

**Table 2:** Summary of treatment-related adverse events

	Preoperative chemotherapy (n = 45)		Postoperative chemotherapy (n = 31)			
	Any grade	≥ Grade 3	Step 1 (n = 11)		Step 2 (n = 20)	
			Any grade	≥ Grade 3	Any grade	≥ Grade 3
*Hematological*						
Platelet count decreased	17 (37.8)	0 (0.0)	1 (9.1)	0 (0.0)	9 (45.0)	1 (5.0)
Anaemia	7 (15.6)	1 (2.2)	1 (9.1)	0 (0.0)	2(10.0)	1 (5.0)
Neutrophil count decreased	6 (13.3)	4 (8.9)	3 (27.3)	1 (9.1)	6 (30.0)	2 (10.0)
WBC count decreased	4 (8.9)	1 (2.2)	2 (18.2)	0 (0.0)	3 (15.0)	0 (0.0)
*Non-hematological*						
Peripheral sensory neuropathy	32 (71.1)	0 (0.0)	0 (0.0)	0 (0.0)	9 (45.0)	0 (0.0)
Diarrhoea	27 (60.0)	8 (17.8)	4 (36.4)	1 (9.1)	12 (60.0)	0 (0.0)
Nausea	25 (55.6)	1 (2.2)	2 (18.2)	0 (0.0)	9 (45.0)	0 (0.0)
Decreased appetite	20 (44.4)	2 (4.4)	5 (45.5)	1 (9.1)	10 (50.0)	1 (5.0)
Stomatitis	20 (44.4)	2 (4.4)	1 (9.1)	0 (0.0)	4 (20.0)	0 (0.0)
Constipation	20 (44.4)	0 (0.0)	1 (9.1)	0 (0.0)	1 (5.0)	0 (0.0)
AST increased	13 (28.9)	0 (0.0)	2 (18.2)	0 (0.0)	6 (30.0)	0 (0.0)
ALT increased	12 (26.7)	0 (0.0)	2 (18.2)	0 (0.0)	5 (25.0)	0 (0.0)
Fatigue	9 (20.0)	1 (2.2)	2 (18.2)	0 (0.0)	2 (10.0)	0 (0.0)
Pyrexia	9 (20.0)	0 (0.0)	0 (0.0)	0 (0.0)	2 (10.0)	0 (0.0)
Duodenal perforation	1 (2.2)	1 (2.2)	0 (0.0)	0 (0.0)	0 (0.0)	0 (0.0)
Hypokalaemia	1 (2.2)	0 (0.0)	0 (0.0)	0 (0.0)	4 (20.0)	3 (15.0)
Palmar-plantar erythrodysaesthesia syndrome	0 (0.0)	0 (0.0)	4 (36.4)	0 (0.0)	1 (5.0)	0 (0.0)

Data are n (%). Adverse events that commonly occur are shown. Adverse events were graded according to the Common Terminology Criteria for Adverse Events (version 4.03)

*WBC* white blood cell, *AST* aspartate aminotransferase, *ALT* alanine aminotransferase

The objective response rate in 12 patients with measurable lesions according to RECIST version 1.1 was 50.0% (90% CI 24.5–75.5). One patient without measurable lesions showed progressive disease.

## Surgery

With the exclusion of one patient showing progressive disease, 44 patients underwent surgery (22 distal gastrectomy, 20 total gastrectomy, one total gastrectomy with lower esophagectomy, and one exploratory laparotomy). Forty-three of 45 patients obtained R0 resection (R0 resection rate: 95.6%, 90% CI 86.7–99.2). Of 45 patients, a pathological response was obtained in 28 (62.2%, 90% CI 48.9–74.3). Six (13.3%) patients obtained pCR, including ypT0N0 in 5 (11.1%). Almost complete regression (< 10% of residual tumour) was observed in 13 (28.9%, 90% CI 19.2–41.0) patients (Table 3). Downward changes from clinical to pathological stages was observed in 31 patients (68.8%, 90% CI 55.7–80.1) (supplementary Table 1).

Grade 3 or higher surgery-related complications were uncommon, with grade 4 postoperative bleeding occurring in one patient (Table 4).

**Table 3:** Surgical findings

	Total (N = 44)
*Type of surgery*	
Distal gastrectomy	22 (50)
Total gastrectomy	20 (45)
Total gastrectomy with lower esophagectomy	1 (2)
Exploratory laparotomy	1 (2)
*LN dissection*	
D2	43 (96)
No dissection	1 (2)
*Resection grade*	
R0	43 (96)
R1/R2	0 (0)
No surgery	1 (2)
*Cytology*	
CY0	37 (84)
CY1	1 (2)
NE	6 (14)

Pathological response	Total (N = 45)
*JGC treatment guideline*	
Grade 0	2 (4)
Grade 1a	14 (31)
Grade 1b	9 (20)
Grade 2	13 (29)
Grade 3	6 (13)
NE	1 (2)
*Becker’s criteria*	
> 50%	20 (44)
10–50%	9 (20)
< 10%	13 (29)
NE	3 (7)
*Response*	
pRR*	28 (62)
pCR**	6 (13)
*yp stage*	
ypT0N0	5 (11)

Data are n (%)

*NE* not evaluated, *pRR* pathological response rate, *pCR* pathological complete response rate

*pRR was defined as a ratio of grade 1b-3 for primary tumors

**pCR was defined as the ratio of grade 3 for primary tumors according to the Japanese classification of gastric carcinoma: 3rd English edition

**Table 4:** Surgical complications

	Surgery (n = 44)					
	Any grade	≥ Grade 3	Grade 1	Grade 2	Grade 3	Grade 4
Hemorrhage	1 (2.3)	1 (2.3)	0	0	0	1
Abdominal infection	3 (6.8)	0 (0.0)	1	2	0	0
Thromboembolic event	2 (4.5)	1 (2.3)	1	0	1	0
Pancreatic fistula	0 (0.0)	0 (0.0)	0	0	0	0
Anastomotic leak	0 (0.0)	0 (0.0)	0	0	0	0
Lung infection	0 (0.0)	0 (0.0)	0	0	0	0
Wound infection	0 (0.0)	0 (0.0)	0	0	0	0

Data are n (%). Adverse events were graded according to the Common Terminology Criteria for Adverse Events (version 4.03)

### Postoperative chemotherapy

Among 43 patients undergoing R0 resection, 31 (11 for Step 1, 20 for Step 2) received postoperative chemotherapy (supplementary table 2). Among the patients who received postoperative chemotherapy (n = 31), pathological Stage I, II and III cases were 6 (19.4%), 17 (54.9%), and 8 (25.8%), respectively.

The reasons for not starting postoperative chemotherapy were pCR without lymph node involvement (yp T0N0) (n = 5), not satisfying criteria for postoperative chemotherapy (n = 4), physician’s decision or withdrawal of consent (n = 2), and perforation of the duodenum during preoperative chemotherapy (n = 1).

DLT was not observed for the first 6 patients undergoing Step 1 chemotherapy. Satisfying the prespecified criterion (no more than 5 of the first 10 patients developing DLT), the study proceeded to Step 2. None of the first 10 patients in Step 2 developed DLT, resulting in the enrolment of a total of 20 patients. Thereafter, 5 additional patients (total n = 11) were enrolled in Step 1. Finally, DLT was neither observed in the evaluable population in Step 1 (n = 10) nor in Step 2 (n = 19). Two patients (one from each step) were not evaluable for DLT because of discontinuation of postoperative chemotherapy for reasons other than AEs.

The completion rates of patients undergoing postoperative chemotherapy in Steps 1 and 2 were 90.9% (10/11) (90% CI 63.6–99.5) and 80.0% (16/20) (90% CI 59.9–92.9), respectively. The reasons for discontinuation of postoperative chemotherapy were patient refusal (n = 1) in Step 1, and physician’s decision (n = 3) and patient refusal (n = 1) in Step 2. Differences in the surgical procedure, such as total gastrectomy (TG) and distal gastrectomy (DG), did not affect the completion rates (completion rate: 85.7% (6/7) with TG and 75.0% (3/4) with DG in Step 1 and 75.0% (6/8) with TG and 83.3% (10/12) with DG in Step 2).

The median RDI of TAS-118 in Step 1 was 83.3%, and the median RDIs of TAS-118 and oxaliplatin in Step 2 were 69.9% and 74.3%, respectively. Grade 3–4 AEs in 11 Step 1 patients were as follows: neutrophil count decreased, decreased appetite, and diarrhoea (each, n = 1 [9.1%]). Grade 3–4 AEs in 20 Step 2 patients were as follows: hypokalaemia (n = 3, [15.0%]), decreased neutrophil count (n = 2, [10.0%]), platelet count decreased, anaemia, and decreased appetite (each, n = 1 [5.0%]) (Table 2).

### Long-term efficacy

**Fig. 3:**
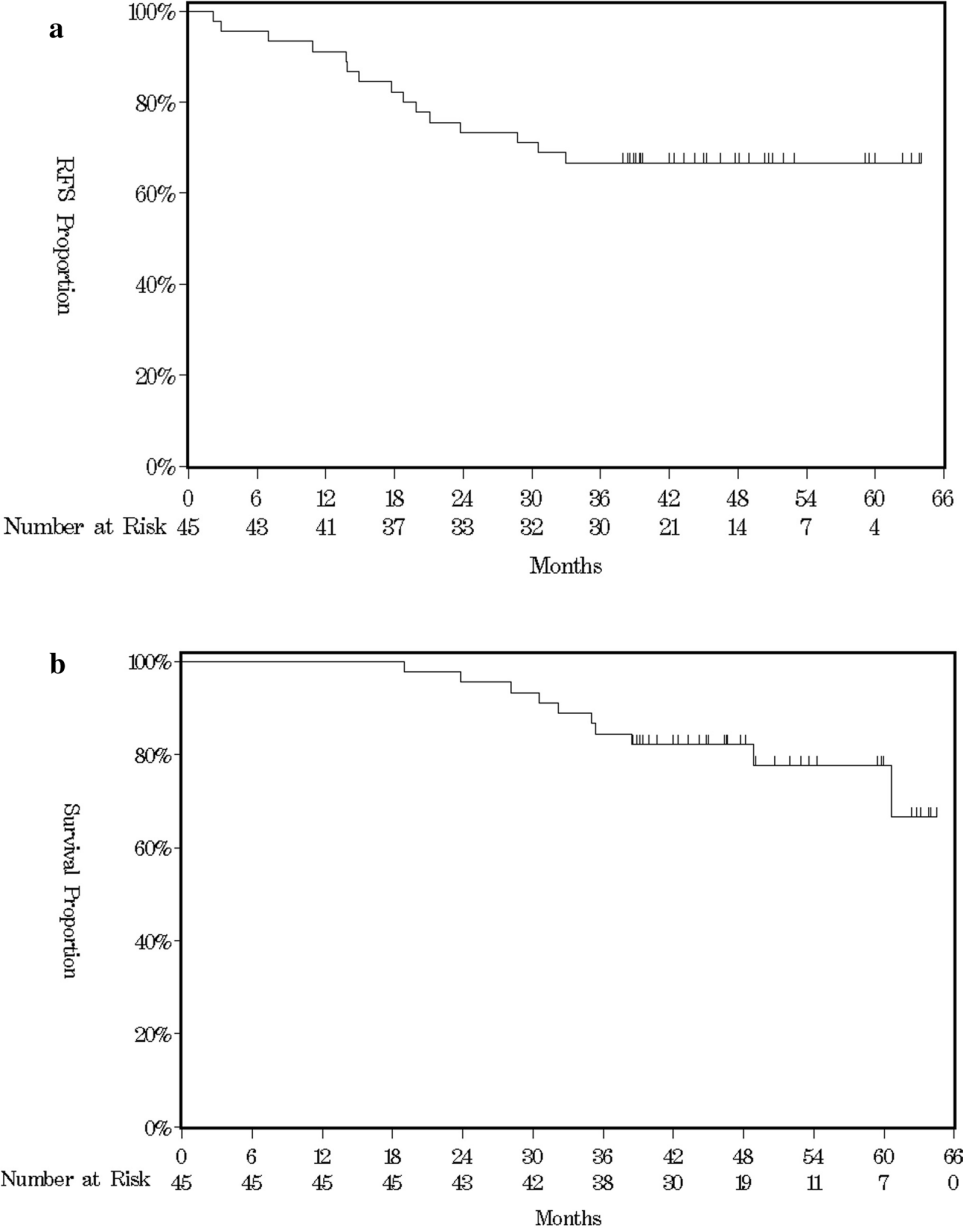
Kaplan–Meier survival curves for **a** progression-free survival, **b** overall survival

As of July 2022 (3.5 years since the last patient was enrolled) 15 of 45 patients developed recurrences and 10 patients died, for a 3-year RFS rate of 66.7% (95% CI 50.9–78.4, Fig. 3a) and 3-year OS rate of 84.4% (95% CI 70.1–92.3, Fig. 3b).

Among the 31 patients who received postoperative chemotherapy, the 3-year RFS rates were 63.6% (95% CI 29.7–84.5) in Step 1 (n = 11, TAS-118 monotherapy) and 80.0% (95% CI 55.1–92.0) in Step 2 (n = 20, TAS-118 plus oxaliplatin), respectively (supplementary Fig. 1). Five patients who obtained ypT0N0 did not receive postoperative chemotherapy, but none of them experienced recurrence (duration of follow-up: 39.4–59.1 months).

## Discussion

This study showed that perioperative TAS-118 plus oxaliplatin followed by D2 gastrectomy for patients with LAGC showed good feasibility. The completion rate of patients receiving preoperative TAS-118 plus oxaliplatin was 88.9% (90% CI 78.0–95.5), and the completion rates of patients receiving postoperative TAS-118 monotherapy and TAS-118 plus oxaliplatin were 90.9% (90% CI 63.6–99.5) and 80.0% (90% CI 59.9–92.9), respectively, which met the primary endpoints of this study, as all lower CI limits were above the thresholds.

Fluoropyrimidine and platinum are the standard regimens for gastric and GEJ cancers. A recent study comparing preoperative chemotherapy regimens found that a triplet regimen that added docetaxel to the doublet therapy of fluoropyrimidine and platinum showed favorable efficacy over doublet chemotherapy alone [5, 6]. However, in Japan, it remains unclear whether preoperative chemotherapy can improve survival, because neither a phase 3 study (JCOG 0501) that evaluated preoperative SP added to D2 surgery plus postoperative S-1 for patients with type 4 or large type 3 GCs [15] nor a phase 2 study (JCOG 1002) that evaluated preoperative docetaxel, cisplatin plus S-1 (DCS) for patients with LAGC with extensive metastatic involvement of the lymph nodes [16] showed a benefit. Moreover, the efficacy and safety of the FLOT regimen has never been reported in an Asian population; therefore, there is no evidence that FLOT is feasible as perioperative chemotherapy for Asian patients. A phase 3 study (JCOG1509) evaluating SOX as preoperative chemotherapy for patients with T3–4 and N1–3 LAGC is now underway [17].

The safety profile of TAS-118 and oxaliplatin used as preoperative chemotherapy in the current study was found to be generally consistent with those reported in previous studies. Although all patients experienced AEs, the incidence of grade ≥3 AEs, including diarrhoea and neutropenia, during preoperative chemotherapy, was less than 20%. These AEs were mostly managed by dose modifications of TAS-118 and/or oxaliplatin or delays in treatment. Furthermore, the completion rate for gastrectomy was high (95.6%), and there were few serious postoperative complications. These results indicate that preoperative TAS-118 plus oxaliplatin did not adversely impact surgery.

The completion rate of postoperative chemotherapy appeared acceptable. While the completion rate was 57.8% (26/45: 90% CI 45.6–69.1%) for all enrolled patients, it was 65.0% (26/40: 90% CI 52.0–76.1%) excluding the 5 patients who did not receive postoperative chemotherapy due to achieving ypT0N0. This did not appear to be inferior to the completion rate of FLOT in the FLOT4 study (47%: 95% CI 40.4–50.7%) [5], those of S-1 monotherapy in the PRODIGY study (63.9%: 95% CI 58.0–69.4%) [6] and the ACTS-GC study (65.8%) [18], and those of postoperative doublet chemotherapy (60–80%) in Asian clinical trials [14, 19, 20]. Moreover, DLT was not observed for patients receiving postoperative chemotherapy consisting of either TAS-118 monotherapy or TAS-118 plus oxaliplatin. Therefore, it is considered that both TAS-118 monotherapy and TAS-118 plus oxaliplatin are feasible for postoperative chemotherapy.

The pathological response of patients with LAGC who undergo chemotherapy is correlated with survival [21]. Terashima et al. recently reported that the pathological response might be used as a surrogate endpoint for studies on preoperative chemotherapy [22]. Although the sample size of this study was small, the pCR rate of 13.3% was higher than that of doublet therapy with SOX (5.6%) [7] and seems comparable to those of triplet chemotherapy with FLOT (13.5%) [5] and DOS (10.4%) [6]. None of the 5 patients with ypT0N0 developed recurrence, even without postoperative chemotherapy. Although pCR may be associated with favorable outcomes in gastric cancer, it is not confirmed from the results of this study that postoperative chemotherapy can be omitted for patients achieving pCR. Moreover, downward changes from clinical to pathological stages was observed approximately 70% of patients, however, given the inaccurate aspects of clinical staging [23, 24], it cannot be an appropriate measure to evaluate the efficacy of preoperative chemotherapy.

The 3-year RFS and OS rates of our study patients, 66.7% (95% CI 50.9–78.4) and 84.4% (95% CI 70.1–92.3), respectively, appeared noninferior to the rates for triplet therapy (46% [95% CI, not stated]) and 57% [95% CI 52–62]), respectively, in the FLOT study [5] and 66.3% (95% CI 59.6–72.1) and 73.4% (95% CI 67.0–78.7), respectively, in the DOS study [6]). Our 3-year RFS and OS rates also seem noninferior to the rates in previous Asian phase 2 studies using perioperative doublet or triplet therapy [25–27], though comparisons with other trials should be made with caution. Notably, for 31 of our study patients undergoing postoperative chemotherapy, the 3-year RFS rate (80.0%) for the postoperative TAS-118 plus oxaliplatin was much higher than the 3-year RFS rate (60.6%) for the postoperative TAS-118 monotherapy.

This study has several limitations. First, it was a relatively small non-randomized feasibility study, and the sample size was not reset to confirm the feasibility and tolerability of postoperative chemotherapy. As a result, only 31 of 45 cases underwent postoperative chemotherapy. However, the feasibility of postoperative chemotherapy in each step was highly suggested by the high completion rate in patients who initiated postoperative chemotherapy.

Secondly, TAS-118 is no longer available because Taiho Pharmaceutical Co., Ltd. has stopped its therapeutic development and discontinued its production. Given the results of this study, we propose that the two components of TAS-118, S-1 and LV [8], should be studied in a larger population. In addition, because the S-1, oxaliplatin and LV (SOL) regimen is doublet chemotherapy, and its associated myelosuppression was mild, it is possible that docetaxel could be added to the SOL regimen. In the future, triplet chemotherapy with SOL + docetaxel could be evaluated as preoperative chemotherapy. Results of a recent randomized phase 2 study indicated that blockade of the programmed cell death protein 1 may improve the pathological response to perioperative chemotherapy for patients with gastric or GEJ cancer [28].There are now some ongoing phase 3 studies evaluating the addition of nivolumab [29], pembrolizumab [30], or durvalumab [31] to a cytotoxic agent for perioperative chemotherapy. The combination of S-1 plus LV and oxaliplatin plus an immune checkpoint inhibitor is a candidate treatment for future development.

In conclusion, perioperative chemotherapy with TAS-118 plus oxaliplatin with D2 gastrectomy is feasible.

## Supplementary Information

Below is the link to the electronic supplementary material.

Supplementary file1 (DOCX 31 KB)

Supplementary file2 (DOCX 34 KB)

Supplementary Fig. 1 Progression-free survival according to postoperative chemotherapy. Step 1: postoperative TAS-118 monotherapy, Step 2: postoperative TAS-118 plus oxaliplatin (PNG 27 KB)

## Data Availability

The data generated and analyzed in this study are available from the corresponding author upon reasonable request.

## References

[1] Bray F, Ferlay J, Soerjomataram I, Siegel RL, Torre LA, Jemal A (2018). Global cancer statistics 2018: GLOBOCAN estimates of incidence and mortality worldwide for 36 cancers in 185 countries. CA Cancer J Clin.

[2] Smyth EC, Verheij M, Allum Wet, Cunningham D, Cervantes A, Arnold D; ESMO Guidelines Committee. Gastric cancer: ESMO clinical practice guidelines for diagnosis, treatment and follow-up. Ann Oncol. 2016;27(suppl 5):v38–49. 10.1093/annonc/mdw350 27664260

[3] Association JGC (2021). Japanese gastric cancer treatment guidelines 2018 (5th edition). Gastric Cancer.

[4] Takahari D, Hamaguchi T, Yoshimura K, Katai H, Ito S, Fuse N (2011). Feasibility study of adjuvant chemotherapy with S-1 plus cisplatin for gastric cancer. Cancer Chemother Pharmacol.

[5] Al-Batran SE, Homann N, Pauligk C, Goetze TO, Meiler J, Kasper S (2019). Perioperative chemotherapy with fluorouracil plus leucovorin, oxaliplatin, and docetaxel versus fluorouracil or capecitabine plus cisplatin and epirubicin for locally advanced, resectable gastric or gastro-oesophageal junction adenocarcinoma (FLOT4): a randomized, phase 2/3 trial. Lancet.

[6] Kang YK, Yook JH, Park YK, Lee JS, Kim YW, Kim JY (2021). PRODIGY: a phase III study of neoadjuvant docetaxel, oxaliplatin, and S-1 plus surgery and adjuvant S-1 versus surgery and adjuvant S-1 for resectable advanced gastric cancer. J Clin Oncol.

[7] Zhang X, Liang H, Li Z, Xue Y, Wang Y, Zhou Z, RESOLVE Study Group, et al. Perioperative or postoperative adjuvant oxaliplatin with S-1 versus adjuvant oxaliplatin with capecitabine in patients with locally advanced gastric or gastro-oesophageal junction adenocarcinoma undergoing D2 gastrectomy (RESOLVE): an open-label, superiority and non-inferiority, phase 3 randomized controlled trial. Lancet Oncol. 2021;22:1081–92. 10.1016/S1470-2045(21)00297-7 34252374

[8] Ioka T, Ueno M, Ueno H, Park JO, Chang HM, Sasahira N (2019). TAS-118 (S-1 plus Leucovorin) versus S-1 in patients with gemcitabine-refractory advanced pancreatic cancer: a randomized, open-label, phase 3 study (GRAPE trial). Eur J Cancer.

[9] Advanced Colorectal Cancer Meta-Analysis Project (1992). Modulation of 5-fluorouracil by leucovorin in patients with advanced colorectal cancer: evidence in terms of response rate. J Clin Oncol.

[10] Thirion P, Michiels S, Pignon JP, Buyse M, Braud AC, Carlson RW (2004). Modulation of fluorouracil by leucovorin in patients with advanced colorectal cancer: an updated meta-analysis. J Clin Oncol.

[11] Kang YK, Chin K, Chung HC, Kadowaki S, Oh SC, Nakayama N (2020). S-1 plus leucovorin and oxaliplatin versus S-1 plus cisplatin as first-line therapy in patients with advanced gastric cancer (SOLAR): a randomized, open-label, phase 3 trial. Lancet Oncol.

[12] Japanese Gastric Cancer Association. Japanese classification of gastric carcinoma: 3rd English edition. Gastric Cancer. 2011;14:101–12. 10.1007/s10120-011-0041-5 21573743

[13] Becker K, Mueller JD, Schumacher C, Ott K, Fink U, Busch R (2003). Histomorphology and regression grading in gastric carcinoma treated with neoadjuvant chemotherapy. Cancer.

[14] Bang YJ, Kim YW, Yang HK, Chung HC, Park YK, Lee KH (2012). Adjuvant capecitabine and oxaliplatin for gastric cancer after D2 gastrectomy (CLASSIC): a phase 3 open-label, randomized controlled trial. Lancet.

[15] Iwasaki Y, Terashima M, Mizusawa J, Katayama H, Nakamura K, Katai H (2021). Gastrectomy with or without neoadjuvant S-1 plus cisplatin for type 4 or large type 3 gastric cancer (JCOG0501): an open-label, phase 3, randomized controlled trial. Gastric Cancer.

[16] Ito S, Sano T, Mizusawa J, Takahari D, Katayama H, Katai H (2017). A phase II study of preoperative chemotherapy with docetaxel, cisplatin, and S-1 followed by gastrectomy with D2 plus para-aortic lymph node dissection for gastric cancer with extensive lymph node metastasis: JCOG1002. Gastric Cancer.

[17] Mizusawa J, Tokunaga M, Machida N, Yabusaki H, Kawabata R, Imamura H (2022). Protocol digest of a phase III trial to evaluate the efficacy of preoperative chemotherapy with S-1 plus oxaliplatin followed by D2 gastrectomy with postoperative S-1 in locally advanced gastric cancer: Japan Clinical Oncology Group study JCOG1509 (NAGISA Trial). Jpn J Clin Oncol..

[18] Sakuramoto S, Sasako M, Yamaguchi T, Kinoshita T, Fujii M, Nashimoto A (2007). Adjuvant chemotherapy for gastric cancer with S-1, an oral fluoropyrimidine. N Engl J Med.

[19] Yoshida K, Kodera Y, Kochi M, Ichikawa W, Kakeji Y, Sano T (2019). Addition of docetaxel to oral fluoropyrimidine improves efficacy in patients with stage III gastric cancer: interim analysis of JACCRO GC-07, a randomized controlled trial. J Clin Oncol.

[20] Shitara K, Chin K, Yoshikawa T, Katai H, Terashima M, Ito S (2017). Phase II study of adjuvant chemotherapy of S-1 plus oxaliplatin for patients with stage III gastric cancer after D2 gastrectomy. Gastric Cancer.

[21] Becker K, Langer R, Reim D, Novotny A, Meyer zumBuschenfelde C, Engel J (2011). Significance of histopathological tumor regression after neoadjuvant chemotherapy in gastric adenocarcinomas: a summary of 480 cases. Ann Surg.

[22] Terashima M, Mizusawa J, Katayama H, Iwasaki Y, kawashima Y, Kinoshita T, et al. Surrogate indicators of survival in patients who received neoadjuvant chemotherapy for type 4 and large type 3 gastric cancer in JCOG0501. J Clin Oncol. 2020;38(suppl 4):abstr 381.

[23] Fukagawa T, Katai H, Mizusawa J, Nakamura K, Sano T, Terashima M (2018). A prospective multi-institutional validity study to evaluate the accuracy of clinical diagnosis of pathological stage III gastric cancer (JCOG1302A). Gastric Cancer.

[24] Kim HD, Lee JS, Yook JH, Ryu MH, Park YK, Kim JY (2022). Radiological criteria for selecting candidates for neoadjuvant chemotherapy for gastric cancer: an exploratory analysis from the PRODIGY study. Gastric Cancer.

[25] Yu Y, Fang Y, Shen Z, Wang Y, Yan M, Cao H (2019). Oxaliplatin plus capecitabine in the perioperative treatment of locally advanced gastric adenocarcinoma in combination with D2 gastrectomy: NEO-CLASSIC study. Oncologist.

[26] Hayashi T, Yoshikawa T, Sakamaki K, Nishikawa K, Fujitani K, Tanabe K (2020). Primary results of a randomized two-by-two factorial phase II trial comparing neoadjuvant chemotherapy with two and four courses of cisplatin/S-1 and docetaxel/cisplatin/S-1 as neoadjuvant chemotherapy for advanced gastric cancer. Ann Gastroenterol Surg.

[27] Iwatsuki M, Orita H, Kobayashi K, Hidaka S, Arigami T, Kusumoto T (2022). Phase II study of S-1 and oxaliplatin as neoadjuvant chemotherapy for locally advanced adenocarcinoma of the gastric or esophagogastric junction: KSCC1601. Gastric Cancer.

[28] Al-Batran SE, Lorenzen S, Thuss-Patience PC, Homann N, Schenk M, Lindig U, et al. Surgical and pathological outcome, and pathological regression, in patients receiving perioperative atezolizumab in combination with FLOT chemotherapy versus FLOT alone for resectable esophagogastric adenocarcinoma: Interim results from DANTE, a randomized, multicenter, phase IIb trial of the FLOT-AIO German Gastric Cancer Group and Swiss SAKK. J Clin Oncol. 2022;40(16_suppl):4003.

[29] Terashima M, Kim YW, Yeh TS, Chung HC, Chen JS, Boku N (2017). ATTRACTION-05 (ONO-4538-38/BMS CA209844): a randomized, multicenter, double-blind, placebo-controlled Phase 3 study of Nivolumab (Nivo) in combination with adjuvant chemotherapy in pStage III gastric and esophagogastric junction(G/EGJ) cancer. Ann Oncol.

[30] Bang YJ, Van Cutsem E, Fuchs CS, Ohtsu A, Tabernero J, Ilson DH (2019). KEYNOTE-585: phase III study of perioperative chemotherapy with or without pembrolizumab for gastric cancer. Future Oncol.

[31] Janjigian YY, Van Cutsem E, Muro K*,* Wainberg ZA, Al-Batran SE, Hyung WJ, et al. MATTERHORN: Efficacy and safety of neoadjuvant-adjuvant durvalumab and FLOT chemotherapy in resectable gastric and gastroesophageal junction cancer—a randomized, double-blind, placebo-controlled, phase 3 study. J Clin Oncol. 2021;39(15_suppl):TPS4151.

