# Supplementary material for: Perioperative TAS-118 plus oxaliplatin in patients with locally advanced gastric cancer: APOLLO-11 study
Source: Gastric Cancer. 2023 Apr 8;26(4):614–25. doi: 10.1007/s10120-023-01388-z (PMC10285008; doi:10.1007/s10120-023-01388-z)
Supplement: Supplementary file 1 — Supplementary file1 (DOCX 31 KB) [file 10120_2023_1388_MOESM1_ESM.docx]

**Supplementary Table 1** Changes in clinical and pathological stages

|  | | Pathological stage | | | | | | | | | |
| --- | --- | --- | --- | --- | --- | --- | --- | --- | --- | --- | --- |
| Clinical stage |  | 0* | IA | IB | IIA | IIB | IIIA | IIIB | IIIC | Ⅳ | n |
|  | IIB | 3 | 0 | 2 | 2 | 5 | 0 | 0 | 0 | 0 | 12 |
|  | IIIA | 2 | 0 | 1 | 3 | 5 | 2 | 1 | 0 | 1 | 15 |
|  | IIIB | 0 | 2 | 2 | 1 | 2 | 4 | 1 | 2 | 1 | 15 |
|  | IIIC | 0 | 0 | 0 | 1 | 0 | 1 | 0 | 0 | 1 | 3 |
|  | n | 5 | 2 | 5 | 7 | 12 | 7 | 2 | 2 | 3 | 45 |

The shaded area shows downgraded cases. *ypT0N0
