# Supplementary material for: Perioperative TAS-118 plus oxaliplatin in patients with locally advanced gastric cancer: APOLLO-11 study
Source: Gastric Cancer. 2023 Apr 8;26(4):614–25. doi: 10.1007/s10120-023-01388-z (PMC10285008; doi:10.1007/s10120-023-01388-z)
Supplement: Supplementary file 2 — Supplementary file2 (DOCX 34 KB) [file 10120_2023_1388_MOESM2_ESM.docx]

**Supplementary Table 2** Characteristics of patients who received/not received postoperative chemotherapy

|  | Postoperative chemotherapy | |
| --- | --- | --- |
|  | Not Administered | Administered |
|  | Total (N=14) | Total (N=31) |
| **Age** |  |  |
| Median [range] | 64 [33-74] | 64 [32-78] |
|  |  |  |
| **Gender** |  |  |
| Male | 11 (78·6) | 26 (83·9) |
| Female | 3 (21·4) | 5 (16·1) |
|  |  |  |
| **ECOG PS** |  |  |
| 0 | 14 (100·0) | 24 (77·4) |
| 1 | 0 (0·0) | 7 (22·6) |
|  |  |  |
| **Primary site**, |  |  |
| Gastric | 11 (78·6) | 29 (93·5) |
| EGJ | 3 (21·4) | 2 (6·5) |
|  |  |  |
| **Histology** |  |  |
| Intestinal | 9 (64·3) | 22 (71·0) |
| Diffuse | 5 (35·7) | 9 (29·0) |
|  |  |  |
| **Clinical T stage** |  |  |
| T3 | 5 (35·7) | 8 (25·8) |
| T4a | 9 (64·3) | 23 (74·2) |
|  |  |  |
| **Clinical N stage** |  |  |
| N1 | 10 (71·4) | 15 (48·4) |
| N2 | 3 (21·4) | 14 (45·2) |
| N3 | 1 (7·1) | 2 (6·5) |
|  |  |  |
| **Clinical stage** |  |  |
| IIB | 4 (28·6) | 7 (22·6) |
| IIIA | 7 (50·0) | 9 (29·0) |
| IIIB | 2 (14·3) | 13 (41·9) |
| IIIC | 1 (7·1) | 2 (6·5) |
|  |  |  |
| **Pathological stage** |  |  |
| 0 | 0 (0·0) | 0 (0·0) |
| IA | 0 (0·0) | 2 (6·5) |
| IB | 1 (7·7) | 4 (12·9) |
| IIA | 0 (0·0) | 7 (22·6) |
| IIB | 2 (15·4) | 10 (32·3) |
| IIIA | 2 (15·4) | 5 (16·1) |
| IIIB | 1 (7·7) | 1 (3·2) |
| IIIC | 0 (0·0) | 2 (6·5) |
| IV | 2 (15·4) | 0 (0·0) |
| Unclassified* | 5 (38·5) | 0 (0·0) |
| NE | 1 (7·7) | 0 (0·0) |
| Data are n (%) or median (IQR). ECOG PS= Eastern Cooperative Oncology Group performance status. EGJ = Esophagogastric junction. NE=not evaluated. *ypT0N0 | | |
