# Supplementary figures and images for: Perioperative TAS-118 plus oxaliplatin in patients with locally advanced gastric cancer: APOLLO-11 study
Source: Gastric Cancer. 2023 Apr 8;26(4):614–25. doi: 10.1007/s10120-023-01388-z (PMC10285008; doi:10.1007/s10120-023-01388-z)

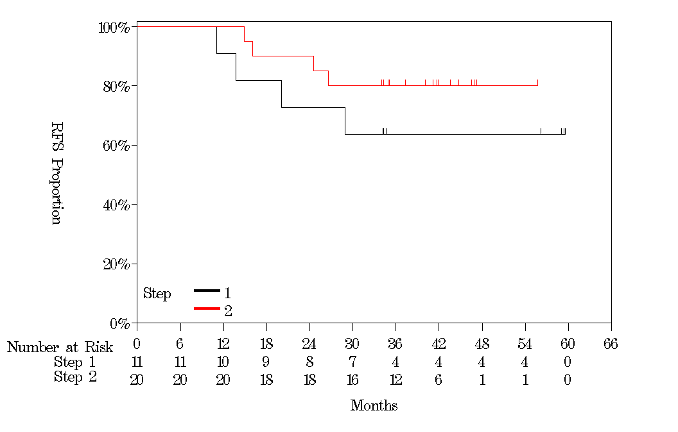

Supplement: Supplementary file 3 — Supplementary Fig. 1 Progression-free survival according to postoperative chemotherapy. Step 1: postoperative TAS-118 monotherapy, Step 2: postoperative TAS-118 plus oxaliplatin (PNG 27 KB) [file 10120_2023_1388_MOESM3_ESM.png]
